# 

**Published:** 1882-06-20

**Authors:** 


Entered at the Post-Office at Atlanta as second-class matter.

VOL. XII. JUNE 20, 1882. NO. 6.

S.G.O. INDEXED

### THE SOUTHERN MEDICAL RECORD:

### A MONTHLY JOURNAL OF PRACTICAL MEDICINE.

Terms: $2 00 per Annum, in Advance; 4 Copies $6.00; Single Copies 20 Cents.

*"QUICQUID PRÆCIPIES ESTO BREVIS."*

